# Helium range viability for online range probing in mixed carbon–helium beams

**DOI:** 10.1002/mp.70194

**Published:** 2025-12-13

**Authors:** Jennifer Josephine Hardt, Alexander A. Pryanichnikov, Oliver Jäkel, Joao Seco, Niklas Wahl

**Affiliations:** ^1^ Division of Medical Physics in Radiation Oncology German Cancer Research Center – DKFZ Heidelberg Germany; ^2^ Heidelberg Institute for Radiation Oncology and National Center for Radiation Research in Oncology Heidelberg Germany; ^3^ Faculty of Physics and Astronomy Heidelberg University Heidelberg Germany; ^4^ Division of Biomedical Physics in Radiation Oncology German Cancer Research Center – DKFZ Heidelberg Germany; ^5^ Institute of Biomedical Engineering (IBT) Karlsruhe Institute of Technology (KIT) Karlsruhe Germany; ^6^ Heidelberg Ion‐Beam Therapy Center Department of Radiation Oncology Heidelberg University Hospital Heidelberg Germany

**Keywords:** carbon therapy, helium imaging, mixed beam, range verification, range shifter

## Abstract

**Background:**

Recently, mixed carbon–helium beams were proposed for range verification in carbon ion therapy: helium, with three times the range of carbon, serves as an online range probe and is mixed into a therapeutic carbon beam.

**Purpose:**

Treatment monitoring is of special interest for lung cancer therapy; however, the helium range might not always be sufficient to exit the patient distally. Therefore, mixed beam use cases of several patient sites are considered.

**Methods:**

An extension to the open‐source planning toolkit, matRad, allows for calculation and optimization of mixed beam treatment plans. The use of the mixed beam method in 15 patients with lung cancer, as well as in a prostate and liver case, for various potential beam configurations was investigated. Planning strategies to optimize the residual helium range considering the sensitive energy range of the imaging detector were developed. A strategy involves adding helium to energies whose range is sufficient. Another one is to use range shifters to increase the beam energy and thus helium range.

**Results:**

In most patient cases, the residual helium range of at least one spot is too low. All investigated planning strategies can be used to ensure a high enough helium range while still keeping a low helium dose and a satisfactory total mixed carbon–helium beam dose. The use of range shifters allows for the detection of more spots.

**Conclusion:**

The mixed beam method shows promising results for online monitoring. The use of range shifters ensures a high enough helium range and more detectable spots, allowing for a wider‐spread application.

## 1 INTRODUCTION

Mixed beams have recently been proposed for range‐guided particle therapy.^1^, ^2^ Hereby two ion species with similar mass‐to‐charge ratios, for example, fully ionized carbon (^12^C^6+^) and helium (^4^He^2+^) ions, are accelerated to a similar energy per nucleon in a synchrotron‐based accelerator. The offset between the mass‐to‐charge ratios is,

(1) $$\chi =\frac{m_{C}}{q_{C}}/\frac{m_{He}}{q_{He}}=0.99935\;.$$

The experimental feasibility of creating such mixed beams was recently demonstrated: Graeff et al.^3^ and Galonska et al.^4^ extracted a mixed beam from a single ion source as ^12^C^3+^, ^4^He^+^, using methane with helium as support gas. Kausel et al.^5^ and Renner et al.^6^ used a double multi‐turn injection, injecting first helium (^4^He^2+^) and then afterwards carbon (^12^C^6+^) into the synchrotron ring.

**FIGURE 1:**
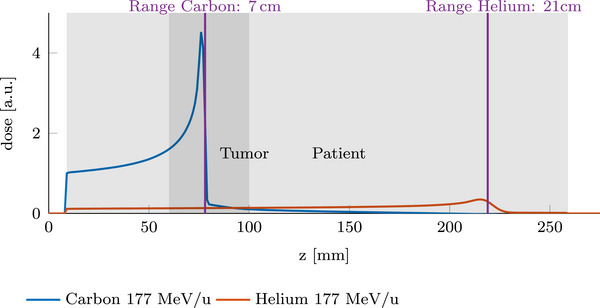
Schematic setup of a mixed carbon–helium irradiation; in this example, the beam energy is not high enough for the helium ions to exit the patient distally.

With carbon and helium ions accelerated to a similar energy per nucleon, the range of helium ions is about three times the range of carbon ions, as shown in Figure 1. Thus, while carbon ions are the primary treatment modality, helium ions, due to their greater range, could exit the patient distally. The residual range of the helium ions could then be measured, offering the potential for online treatment monitoring to verify the water‐equivalent thickness (WET) at the irradiated spot positions. Mazzucconi et al.^1^ and Volz et al.^7^ discussed an additional helium fluence of 10% to ensure a low additional dose to the patient while still achieving a detectable helium signal above the carbon fragment background.

Hardt et al.^8^ demonstrated potential use cases of the mixed carbon‐helium beam method in treatment verification, investigating patient positioning verification or breath‐hold verification. We extended the open‐source treatment planning tool matRad ^9^, ^10^ for mixed beam treatment planning. Including fast pencil beam dose calculation with custom high‐energy helium pencil‐beam kernels and exportation of the treatment plan to the Monte Carlo system, TOPAS,^11^, ^12^ to allow for simulation of helium radiographs.

The pipeline for simulation of helium radiographs presented by Hardt et al.^8^ modeled the proton radiography detector by ProtonVDA.^13^ This system was recently used for helium radiographs,^14^ as well as a feasibility study for intrafractional motion management.^15^ This system utilizes two trackers to measure the positions of the ions, one proximal and one distal to the patient, and a calorimeter distal to the patient to measure the energy. However, with the high carbon intensities used for radiation therapy, the proximal or front tracker is expected to be saturated by the used intensity, thus only a distal tracker is used, which has been shown to reduce the spatial resolution of the resulting image.^16^

**FIGURE 2:**
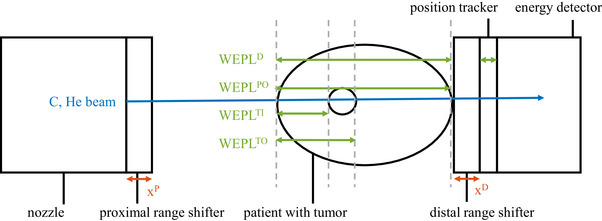
Schematic setup of mixed beam irradiation, showing a proximal and distal range shifter, the detection system, and a visualization of the WEPL thicknesses used in the calculations. WEPL, water equivalent path length.

Taking a closer look at the helium range, Figure 1 shows it to be about three times the carbon range at the same energy per nucleon. However, depending on the tumor site, the helium range may not be sufficient for the ions to exit the patient distally, as also seen in Figure 1. For lung tumors, common irradiation geometries often require only a small carbon range to reach the tumor. This results in a correspondingly short helium range. Complementarily, for more deep‐seated tumors, the high residual helium range of the exiting ions can challenge the sensitive window of the detector. In this work, we investigate the expected residual helium range for several cancer sites, namely prostate, liver, and lung. Next to this, several helium range strategies are proposed to choose and modify the residual helium range: *EW He* (energy‐wise helium), *const RaShi* (constant range shifter), and *EW RaShi* (energy wise range shifter). These strategies will be presented in more detail in the following section and should ensure sufficient helium range to exit the patient distally, but also consider the sensitive range of the used detection system. In short, we propose irradiating only the portion of the energies in the treatment plan with sufficient helium range with a mixed carbon–helium beam (*EW He*). Additionally, we propose adding proximal range shifters to increase the beam energy and distal range shifters to reduce the helium range when necessary. This ensures that the residual helium energy is within the range that the detector can measure (*const RaShi*, *EW RaShi*). A schematic setup of the mixed beam irradiation, including the range shifters and the detection system, is displayed in Figure 2.

During treatment planning, a ray tracing algorithm^17^ calculates the (water equivalent path length) WEPL traversed by the particle beam. This allows us to estimate the residual helium range by subtracting the traversed WEPL from the initial range. The WEPLs at various depths along the ion paths are calculated and used in the following analyses. They are also shown in Figure 2 and will be discussed below.

## 2 MATERIALS AND METHODS

### 2.1 Reconstruction of helium radiographs

The pipeline presented in Hardt et al.^8^ was extended to allow for a simulation without a front tracker, that is, the positions of the ions are only measured distal to the patient. During image reconstruction, the position of the ions on the front tracker is estimated from the beam monitoring system, and a most likely path algorithm is used to calculate the ions path. The passed through WEPL of each helium ion is calculated from its initial and final energy. The WEPL values are grouped into pixels according to the intersection point of the ion's path with the chosen image plane, typically the beam isocenter plane. After applying statistical cuts, the average WEPL for each image pixel is computed.^13^, ^18^, ^19^ During our simulations, the geometry and materials of the tracking detector is simulated, and the phase space of the primary helium ions at the position of the tracker is saved. From this, we reconstruct the images.

### 2.2 Strategies to optimize the residual Helium range

#### 2.2.1 Selection of energies for mixed beam irradiation

The first investigated strategy (*EW He*) avoids helium with insufficient range by using a mixed carbon–helium beam only at energies with a sufficiently high helium range and a pure carbon beam for the remaining energies. The most straightforward implementation of this strategy would be to first irradiate all mixed carbon–helium energy layers within a beam to obtain anatomical information, and then switch to a carbon‐only beam for the remaining energy layers. Alternatively, carbon and mixed carbon–helium energies could be irradiated in an alternating pattern. The method of switching between radiation modes depends on how the mixed beam is generated. This involves either switching ion sources or changing the injection parameters in the case of the double multi‐turn injection method.

To implement this method, the minimum residual helium range is calculated for each energy. If it is smaller than the safety margin $R^{\min}$, the energy is marked for irradiation with a conventional carbon beam. We consider splitting up the irradiation into energy layers with and without sufficient helium range rather than into spots with and without sufficient helium range so as not to prolong the treatment time too much.

#### 2.2.2 Selection of proximal and distal range shifters

Since the helium range is three times the carbon range, the range of the carbon ions within the beam needs to be at least 1/3 of the minimum helium range required for the helium ions to distally exit the patient. To ensure that, a proximal range shifter ($x_{P}$) can be used to adjust the proximal material budget.

Since not every helium energy can be measured by the used detection system, the use of distal range shifters ($x_{D}$), placed between the patient and the detector, is considered. This range shifter lowers, if needed, the residual helium energy at the detection system for optimal detection properties.

We developed an algorithm to automatically choose the WET of the proximal and distal range shifter based on the residual helium range and the sensitive range of the chosen detector. Several discrete options for the WET of the proximal ($O_{P}=\left\{0,5,15,25,35,45\;mm\right\}$), and for the distal range shifter ($O_{D}=\left\{0,10,20,30,40,50,60,70,80,90,100,110,120,130,140,150\;mm\right\}$) were considered. The proximal range shifter thicknesses are chosen to be consistent with range shifters already used in clinics; Wang et al.^20^ used range shifters with thicknesses of up to 41.2mm.

First we calculate which proximal range shifters ($x_{P}$) and energy combinations (E) are possible, that is, if the carbon range ($R_{C}\left(E\right)$) is within the target and if the helium range ($R_{He}\left(E\right)$) is high enough for helium to traverse the entire patient. We calculate the following for all energy and proximal range shifter combinations and only consider combinations for which this holds true.

(2) $$A\left(x^{P},E\right)=\left\{\begin{array}{cc} 1, & \text{if}\;{\text{WEPL}}^{\text{TI}}\leq R_{C}\left(E\right)-x^{P}\leq {\text{WEPL}}^{\text{TO}}\\ & \;\text{and}\;R_{He}\left(E\right)-{\text{WEPL}}^{\text{PO}}-x^{P}\geq R^{\min}\\ 0, & \text{otherwise}. \end{array}\right.$$

Hereby, ${\text{WEPL}}^{\text{TI}}$ and ${\text{WEPL}}^{\text{TO}}$ are the traversed WEPL at the tumor entrance and exit and ${\text{WEPL}}^{\text{PO}}$ the total WEPL of the patient, as also displayed in Figure 2. The safety margin of the minimum allowed residual helium range distal of the patient is $R^{\min}$.

For each of the available combinations, we then estimate if the helium ions will be detectable by our chosen detector, that is, if the residual helium range is in the sensitive window of the detector denoted as $\left[R^{minD},R^{maxD}\right]$. For this we calculate:

(3) $$\begin{array}{cc} & D(x^{P},x^{D},E)\\ = & \left\{\begin{array}{cc} 1, & \text{if}\;R^{\text{minD}}\leq R_{He}\left(E\right)-{\text{WEPL}}^{D}-x^{P}-x^{D}\leq R^{\text{maxD}}\\ 0, & \text{otherwise.} \end{array}\right. \end{array}$$

Whereby, ${\text{WEPL}}^{D}$ is the traversed WEPL at the entrance of the detector. Contrary to ${\text{WEPL}}^{\text{PO}}$ this could include the WEPL of patient couch and of the position tracking system.

Two approaches were considered for selecting the optimal range shifter. In the first method, each treatment field has a single fixed proximal and distal range shifter for all delivered energies (*const RaShi*). In contrast, the second method allows energy‐dependent selection of the proximal range shifter (*EW RaShi*).

The concept behind this approach is that the proximal range shifter attached to the nozzle could be used, enabling a quick selection of a different thickness for different energies. The distal range shifter is positioned directly between the patient and the detector, with as little air as possible between them to reduce scattering, as shown in Figure 2. We consider a constant thickness of the distal range shifter throughout the delivery of the treatment field, a dynamic selection of the distal thickness was not considered. Introducing a device capable of varying the distal thickness would require integrating additional moving components close to the patient, complicating the clinical workflow. In contrast, a fixed distal range shifter, such as a PMMA block in front of the detector, is simple to implement. Therefore, we use the more practical and simpler configuration of a variable proximal range shifter combined with a fixed distal one.

##### Const RaShi

To determine the optimal combination of proximal and distal range shifters for a field, we strive for a high detection percentage while minimizing the WET thickness of the selected range shifters. A greater thickness leads to increased beam broadening and image noise. Therefore, we maximize the weighted sum of the number of detectable spots and the total thickness of the applied range shifters:

(4) $$\begin{array}{cc} \left[x^{P★},x^{D★}\right] & =\underset{\begin{array}{c} x^{P}\in O^{P}\\ x^{D}\in O^{D} \end{array}}{\mathrm{argmax}}\left(\frac{1}{n_{1}}\left(\sum_{s\in F}D\left(x^{P},x^{D},E_{s}\right)\right)\right.\\ & \;-\left.\frac{w}{n_{2}}\left(x^{P}+x^{D}\right)\right). \end{array}$$

The sum is performed for all spots s belonging to the treatment field F. The parameter w serves as a relative weighting factor of the number of detectable spots to the thickness of the range shifters. The factors $n_{1}$ and $n_{2}$ normalize both parts of the function to have equal magnitude

(5) $$n_{1}=\max_{\begin{array}{c} x^{P}\in O^{P}\\ x^{D}\in O^{D} \end{array}}\left(\sum_{s\in F}D\left(x^{P},x^{D},E_{s}\right)\right)$$

(6) $$n_{2}=\max_{\begin{array}{c} x^{P}\in O^{P}\\ x^{D}\in O^{D} \end{array}}\left(x^{P}+x^{D}\right)\;.$$

##### EW RaShi

As a proximal range shifter might not be needed for every energy, or a thinner one is sufficient for higher beam energies, we consider the use of different proximal range shifter thicknesses for different primary beam energies. The selection of the optimal thickness for each energy is more complex for this strategy, since a given depth in the patient can be reached through multiple energy and proximal range shifter combinations. Therefore, we group the spots into intervals ($I_{i}$) of approximately the same carbon Bragg peak position in the patient ($R_{C}^{P}=R_{C}-x^{P}$). The width of each interval is the chosen longitudinal spot spacing l. With this interval, $I_{i}$ is given as

(7) $$I_{i}=\left[\min\left(R_{C}^{P}\right)+il,\min\left(R_{C}^{P}\right)+\left(i+1\right)l\right]\;i=0,1,\cdots ,N.$$

Whereby the last interval $I_{N}$ includes the most distal carbon range position ($\max(R_{C}^{P})$).

For each distal range shifter ($x^{D}$) and carbon range interval ($I_{i}$), the optimal proximal range shifter ($x_{iD}^{P★}$) with corresponding energy ($E_{iD}^{★}$) combination is selected as the one yielding the highest number of detectable spots.

(8) $$\left[x_{iD}^{P★},E_{iD}^{★}\right]=\underset{x^{P}\in O^{P}}{\mathrm{argmax}}\left(\sum_{s\in I_{i}}D\left(x_{s}^{P},x^{D},E_{s}\right)\right).$$

The number of detectable spots for this range interval ($I_{i}$) with optimized settings is $D_{iD}$. Now, since the optimal proximal range shifters with their corresponding beam energy are chosen for each distal range shifter option, it still has to be determined which distal range shifter ($x^{D★}$) with corresponding proximal range shifters should be used. Hereby, the total number of detectable spots is maximized while minimizing the overall thickness of the selected range shifters, similar to the *const RaShi* selection

(9) $$x^{D★}=\underset{x^{D}\in O^{D}}{\mathrm{argmax}}\left(\frac{1}{n_{1}}\left(\sum_{i=0}^{N}D_{iD}\right)-\frac{w}{n_{2}}\left(\sum_{i=0}^{N}x_{iD}^{P★}+x^{D}\right)\right).$$

Whereby the normalization is given as

(10) $$n_{1}=\max_{x^{D}\in O^{D}}\left(\sum_{i=0}^{N}D_{iD}\right)$$

(11) $$n_{2}=\max_{x^{D}\in O^{D}}\left(\sum_{i=0}^{N}x_{iD}^{P★}+x^{D}\right)$$

#### 2.2.3 Incorporating proximal range shifters in dose calculation

To model the additional beam broadening caused by range shifters, Monte Carlo simulations were conducted using TOPAS version 3.9. The simulations employed the following physics list: G4DecayPhysics, G4StoppingPhysics, G4EmExtraPhysics, G4EMStandardPhysics_option4, G4HadronElasticPhysics, g4h‐phy_QGSP_BIC_HP, and G4QMDReaction physics. To accurately model helium ions, G4BinaryLightIonReaction was activated with the Tripathi cross section data^21^ as modified by Horst et al.^22^

The simulations included carbon and helium beams at five different energies within the carbon treatment energy range (88.83,196.23,272.77,339.80and427.44MeV/u). Energy deposition was scored in a cylindrical volume, with a range shifter, composed of PMMA, placed in front. Seven different water equivalent thicknesses of the range shifter were used ($x^{P}$ = 5,10,15,20,25,30,35,40and45mm). The range shifter was positioned at the nozzle and thus separated from the cylinder's surface at the isocenter by approximately 1m of air.

The lateral entrance dose was fitted with a Gaussian function, and the initial beam width was subtracted quadratically to estimate the beam broadening induced by the range shifter and the passed through air ($\sigma_{RaShi}$). A polynomial function was then fitted to interpolate beam broadening at intermediate energies. During dose calculation, the estimated beam broadening from the range shifter is added to the initial beam width.^9^, ^23^

### 2.3 Investigated patient cases

**FIGURE 3:**
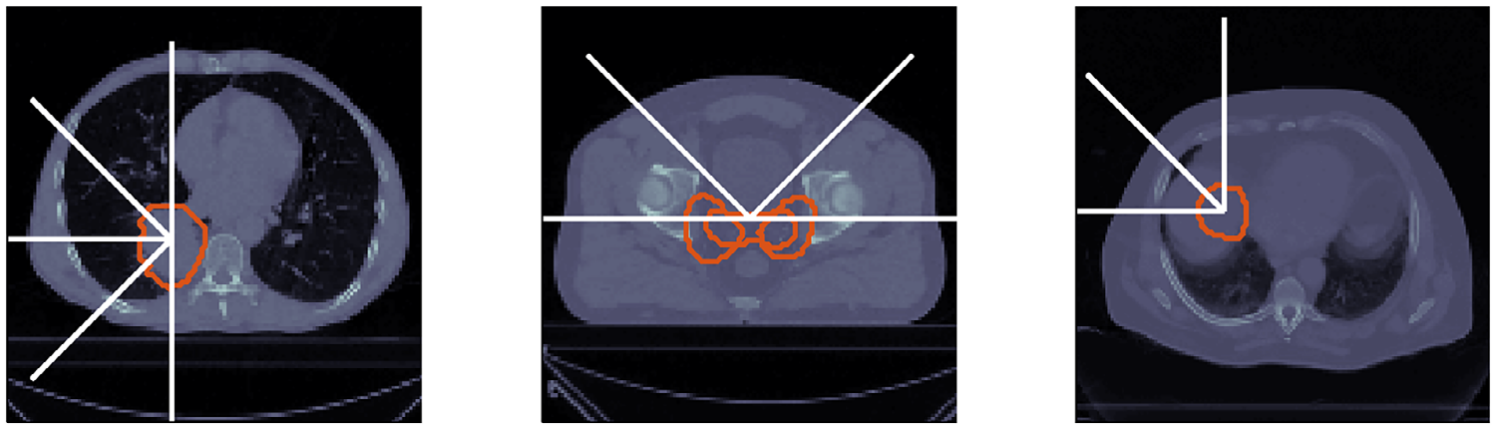
Left: Example lung patient (No. 114) from the lung data set, outlined is the target volume (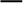) and the gantry angles for which the residual helium range was investigated. Middle: Prostate case. Right: Liver case

We analyzed the residual helium range in 15 lung cancer patient cases,^24^ considering five different gantry angles: 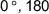, as well as 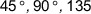 for tumors in the right lung or 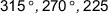 for tumors in the left lung. An example patient is shown in Figure 3.

Additionally, we conducted a detailed analysis of one patient (No. 114) by generating treatment plans for a gantry angle of 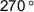 across all residual helium range strategies. The dose influence matrix was calculated using a regular spot grid with lateral spacing of 5mm and a longitudinal spacing of approximately 2mm (depending on available energies) on a $3\;mm^{3}$ dose grid. The RBE‐weighted carbon ion dose (LEM I) was optimized to 2.3Gy per fraction for the target PTV (planning target volume). Helium radiographs for these plans were also simulated using 1e7 histories for the entire field.

Furthermore, we examined the residual range in a prostate cancer patient,^25^ with both a low‐dose PTV (56Gy) and a high‐dose PTV (68Gy), evaluating gantry angles of 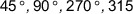. For a liver cancer patient,^25^ we investigated gantry angles of 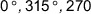.

An overview of the investigated tumor sites and corresponding gantry angles is provided in Figure 3.

### 2.4 Parameter selection

In the presented strategies, there are several parameters that have to be selected. The safety margin of the minimum residual helium range was $R^{\min}=10\;\mathrm{mm}$, as close to the end of the range, the dose can already increase.

If we consider a calorimeter‐like detector, the minimum and maximum values of the detectable range are related to the minimum amount of deposited energy necessary for an accurate measurement and the total WET of the detector. In our investigation, we used a minimum value of $R^{\text{minD}}=7.5\;mm$ based on previous experience with the ProtonVDA^13^ detector. We investigated several values for the maximum detectable range. The smallest investigated value was based on the ProtonVDA detector ($R^{\text{maxD}}=110\;mm$). Further values were 160mm,210mmand260mm, representing detectors with a 5cm,10cmand5cm larger detectable energy range. For the ProtonVDA detector, the WET of the tracker is 5mm.

For the prostate and liver cases, we assigned a relative weighting factor w of 0.25 (mentioned in equation 4), for the lung cases, this weighting factor was set to 0.5. For the lung cases, we anticipate lower residual helium ranges distal of the patient, so thinner distal range shifters are sufficient. As a result, the thickness of the range shifters was less penalized in these cases. In contrast, for the prostate case, located centrally within the patient, we expect significantly higher residual helium ranges, necessitating the use of thicker distal range shifters. To account for this, a smaller penalty weight was applied, placing greater emphasis on maximizing the number of detectable spots.

## 3 RESULTS

### 3.1 Residual range analysis for different cancer sites

#### 3.1.1 Lung cases

**FIGURE 4:**
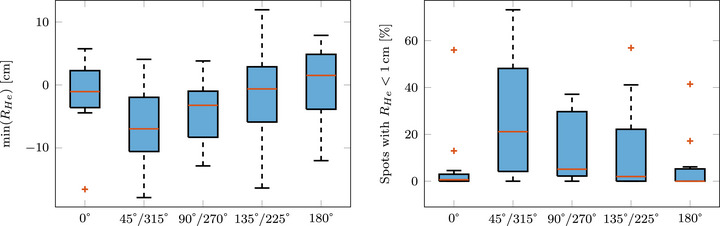
Left: Box plot summarizing the minimum residual helium range of each lung patient for the different gantry angles. Right: Box plot summarizing the percentage of spots in each treatment plan with a residual helium range smaller than 1cm for each gantry angle.

During the investigation of the residual helium range, none of the previously mentioned helium range strategies were applied; instead, conventional treatment plans were created, as the first phase of this study aimed to evaluate the necessity of such strategies. Figure 4 provides an overview of the minimum residual helium range for each investigated gantry angle and patient case, along with the percentage of spots where the residual helium range is less than 1cm. This serves as an indication of the severity, helping to determine whether only a small or large fraction of spots are identified as having insufficient range. Negative values indicate spots where the helium ions lacked sufficient energy to exit the patient distally.

Among the examined angles, 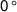 and 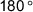 appear to be the most suitable for mixed beam irradiation without additional range strategy, as they offer a higher minimum residual helium range and a lower percentage of spots with insufficient range. However, 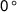 may be preferable to 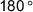 since it avoids irradiation through the patient couch. Despite this, for 9 out of the 15 patients analyzed, the minimum residual helium range for a gantry angle of 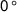 was still below the 1cm safety margin. Furthermore, in 6 of the 15 cases, none of the investigated angles provided sufficient range. The most frequently available angle was 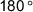 (8/15) followed by 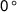 and 135/225 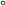 (6/15). The outliers in Figure 4 represent patient cases with particularly unfavorable tumor placements, for example, where the heart is located distal to the tumor, requiring high beam energies for the helium ions to have sufficient range.

**FIGURE 5:**
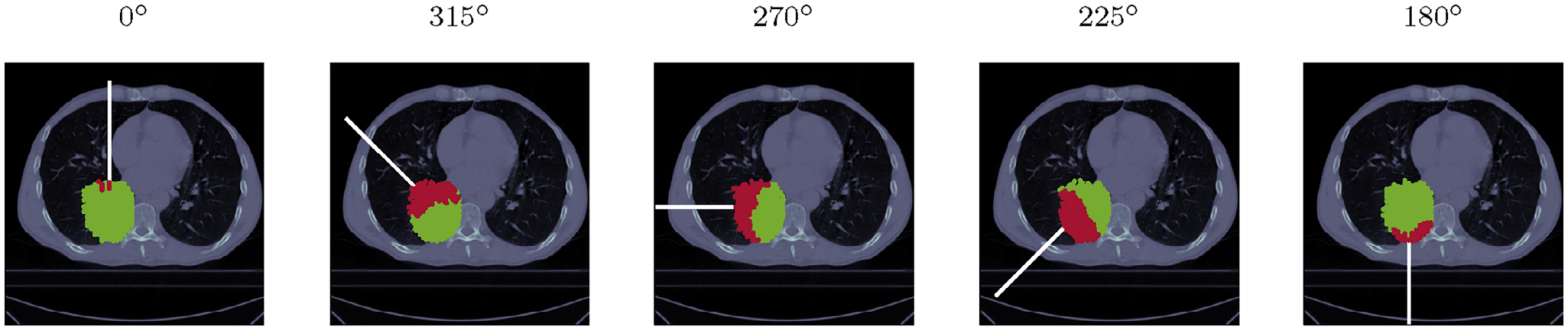
Axial CT slice for patient No.114 for different gantry angles. The overlay highlights the spots with sufficiently large residual helium range in green (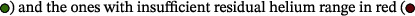) and the ones with insufficient residual helium range in red (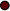).

Figure 5 uses an exemplary lung patient (No. 114) to illustrate the distribution of spots with sufficient and not sufficient helium range. This case exhibits a higher proportion of spots with insufficient helium range compared to the average lung patient. For the 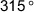 gantry angle, it is particularly evident that the helium beam is most affected in regions where it must pass through the spine.

#### 3.1.2 Prostate case

In the prostate case, the residual helium range was examined for four gantry angles. Compared to the lung case, far fewer spots were affected by insufficient residual helium range. Specifically, only 1.1% of spots exhibited insufficient helium range for the 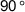 angle, which represented the highest percentage of spots with insufficient helium range among the four investigated angles. For the other angles, the percentages were 0.9% (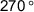), 0.2% (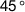), 0% (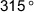). Despite the prostate being centrally located within the patient, which might suggest that residual helium range would not be a concern, it still is for this case.

#### 3.1.3 Liver case

For the liver case, the residual helium range was analyzed for three gantry angles. All three angles exhibited spots with insufficient residual helium range. For the 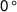 gantry angle, only 1% of the spots were flagged as insufficient, whereas 47% and 38% of spots were flagged as insufficient in the 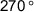 and 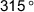 angles, respectively.

### 3.2 Beam broadening due to range shifters

**FIGURE 6:**
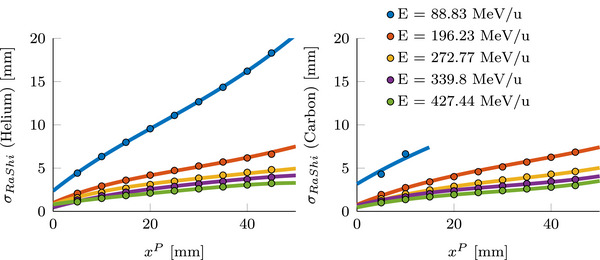
Estimated (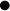) and fitted (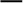) beam widening due to the use of a proximal range shifter of varying thickens for helium (left) and carbon (right).

Figure 6 shows the estimated beam widening $\sigma_{RaShi}$ caused by the use of the proximal range shifter as well as the interpolated function. Especially for low energies and a thick range shifter, the broadening is large. However, this combination is infrequently used in treatment plans. Normally, a thicker range shifter will be used with a higher beam energy. Not for all carbon energies and proximal range shifter combinations was the widening calculated, for the lowest energy, the used range shifters were sometimes thicker than the corresponding carbon range. This would also mean that these energies would not be used in a treatment plan, and thus it is not necessary to calculate the helium widening in these cases; however, this was done for completeness. This beam broadening was used in the following dose calculations.

### 3.3 Strategies to optimize the residual helium range

In general, all residual helium range strategies effectively ensure a sufficient helium range. The strategies are compared based on the percentage of detectable spots. Helium ions may not be detectable for two main reasons: they are lost in the distal range shifter, or their residual energy is too high for the detector to measure.

#### 3.3.1 Lung cases

**FIGURE 7:**
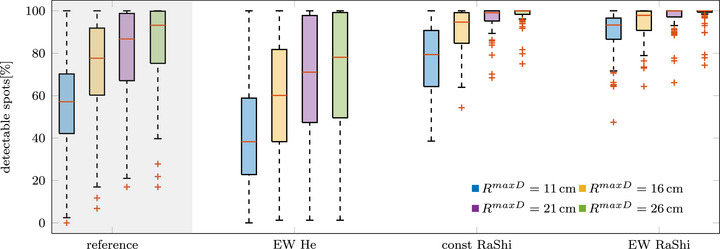
Box plot summarizing the percentage of detectable spots in the lung treatment plans for detectors with varying sensitive ranges ($R^{maxD}=$ 11cm,16cm,21cmand26cm).

Figure 7 presents the percentage of detectable spots across all treatment angles and patients for the presented residual helium range strategies. The calculation was conducted for four imaging detectors, with different sensitive ranges. As a reference, a treatment plan was calculated without applying any residual helium range strategy, meaning that this plan may include spots with an insufficient helium range.

As expected, the detectors with wider acceptable residual helium range allow for a larger percentage of detectable spots. The difference between the detectors is relatively small for the best‐performing method, *EW RaShi* and averages 5 pp ($R^{maxD}=11\;cm\;\;\mathrm{and}\;6\;cm$), 8 pp ($R^{maxD}=11\;cm\;\;\mathrm{and}\;\;21\;cm$), 9 pp ($R^{maxD}=11\;cm\;\;\mathrm{and}\;\;26\;cm$). Among the strategies, *EW He* performs the worst, even falling below the reference plan. When comparing the two range shifter strategies, the added flexibility of the *EW RaShi* method results in an average increase in detectable spots of 12 pp ($R^{maxD}=11\;cm$), 4 pp ($R^{maxD}=16\;cm$), 1 pp ($R^{maxD}=21\;cm$), and 0.2 pp ($R^{maxD}=26\;cm$).

For the detector with the larger sensitive ranges, smaller distal range shifters are used on average, which would enhance image quality. For the *const RaShi*, the thickness of the distal range shifter, if one is used, reduces for the investigated lung cases on average by 2cm ($R^{maxD}=11\;cm\;\;\mathrm{and}\;6\;cm$), 3cm ($R^{maxD}=11\;cm\;\;\mathrm{and}\;\;21\;cm$), and 4cm($R^{maxD}=11\;cm\;\;\mathrm{and}\;\;26\;cm$). For the *EW RaShi*, the thickness of the distal range shifter reduces on average by 4cm ($R^{maxD}=11\;cm\;\;\mathrm{and}\;6\;cm$), 5cm ($R^{maxD}=11\;cm\;\;\mathrm{and}\;\;21\;cm$), and 6cm ($R^{maxD}=11\;cm\;\;\mathrm{and}\;\;26\;cm$).

#### 3.3.2 Lung case No.114

To look at the different strategies in more detail, the delivered dose was optimized and calculated for an example patient. Table 1 lists the percentage of detectable helium spots, and since the optimal fluence of each spot was now calculated, the percentage of detectable helium ions was also calculated. When calculating the percentage of detectable helium ions for the *EW He* method, we did not use the total number of delivered helium ions. Instead, we used 10% of the number of carbon ions—corresponding to the carbon–helium ratio—as a reference value to allow for better comparison between the methods. For the reference and *EW He* strategies, the percentage of detectable helium ions increases, while the percentage of detectable ions decreases for the *const RaShi* strategy when compared to the percentage of detectable spots. Due to the higher fluence of the intermediate energies, which are detectable by the reference plan, the reference and *const RaShi* have the same percentage of detectable helium ions, although the percentage of detectable spots is significantly higher for the *const RaShi* strategy. A range shifter of only 1.5cm (*const RaShi*) already allows the detection of 24 pp more helium ions than with the *EW He* method and as many helium ions as with the reference plan, but with sufficient residual helium range.

**TABLE 1:** Percentage of detectable spots and helium ions in lung case No. 114 for each method.

	Reference	*EW He*	*const RaShi*	*EW RaShi*
Detectable spots (%)	55	35	76	93
Detectable helium ions (%)	69	46	70	93

*Note*: The percentage of detectable helium ions is given relative to 10% of the carbon fluence.

**FIGURE 8:**
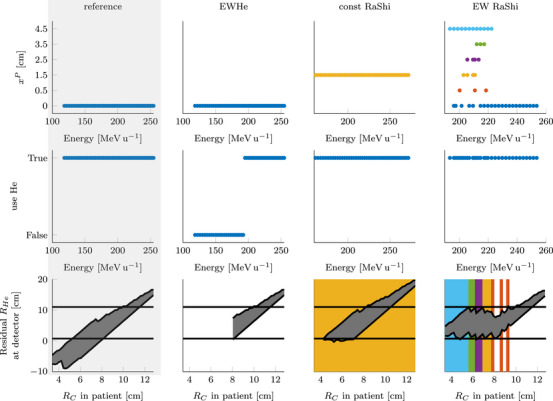
For each strategy (by column) the top row shows the proximal range shifter thickness per energy in the treatment plan. In the middle row illustrates the decision of mixing helium (True) into the carbon beam or not (False) for each energy. The bottom row compares, for each spot, the carbon range with the residual helium range at the detector. Highlighted is the minimum (0.75cm) and maximum (11cm) detectable range, and the used proximal range shifter thickens. Whereby 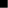 represents a proximal range shifter thickness of 45mm, 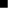 35mm, 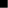 25mm, 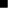 15mm, and 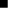 5mm.

Figure 8 provides a closer look at the selected proximal range shifter thickness, which treatment energies used helium, and the last row, which shows the residual helium range at the detector versus the carbon range in the patient, overlaid with the used proximal range shifter thickness. In this lung case, the optimal choice is not to use a distal range shifter. This investigation also employs the smallest detector ($R^{\mathrm{maxD}}=11\;cm$). The *EW RaShi* strategy uses thicker proximal range shifters than the *const RaShi* strategy. Looking at the lower right plot, it is clear that the thickest proximal range shifter is used for the lowest carbon range in the patient and the lowest energy, and then the thickness of the proximal range shifter decreases. Note how the proximal range shifter is used to “push back” the residual helium range into the sensitive range of the imaging detector. So while the *EW RaShi* method has 23 pp more detectable helium ions than the *const RaShi*, it comes at the cost of a thicker proximal range shifter. For the *EW He* strategy, helium is used only with high energies, where the helium range is sufficient. Also, noteworthy for the *const RaShi* method is that the smallest carbon range is not irradiated. These spots must have had too low helium range and were therefore excluded, but a thicker range shifter would have come at the cost of a reduced number of detectable spots.

**FIGURE 9:**
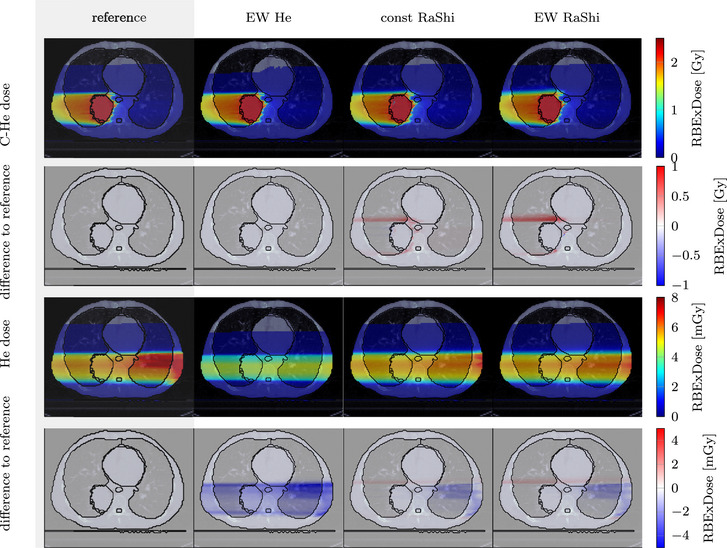
Axial dose slices for each strategy. Top: total mixed carbon‐helium RBE weighted doses and below the difference to the reference plan. Bottom: Helium RBE weighted dose and the difference to the reference plan, please note the mGy scale in this case.

**FIGURE 10:**
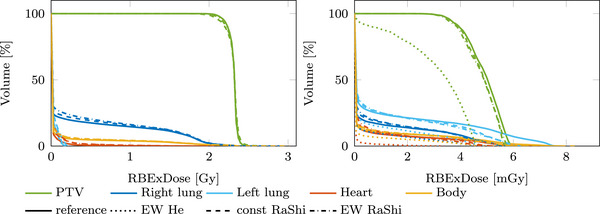
Dose–volume histograms (DVH) for the different strategies. The left DVH shows the total mixed carbon–helium RBE weighted dose, while the right DVH singles out the helium dose (on a mGy scale).

The mixed carbon–helium and helium doses for all strategies are shown in Figure 9 with the respective differences from the reference plan. Figure 10 shows the corresponding carbon–helium and helium dose–volume‐histograms (DVH). The residual helium range strategies reduce the delivered helium dose. This is particularly noticeable for the *EW He* method with a significant reduction in helium dose to the PTV, left lung, heart, and body. However, since the helium dose contributes little to the total dose, the dose reduction is barely visible in the mixed‐dose DVH.

Strategies involving range shifters introduce increased lateral scatter, as visible in Figure 9 as a widened dose profile and resulting increase in dose to the right lung. The mean dose increases from 0.28Gy (reference) to 0.31Gy (*const RaShi*) and 0.33Gy (*EW RaShi*). For these plans, there is also a reduction in target coverage: the $D_{95}$ value of the PTV decreases from 2.19Gy (reference) to 2.13Gy (*const RaShi*) and 2.16Gy (*EW RaShi*). In general, using range shifters increases the total delivered dose of the plans over the reference plan, in this case 10% (*const RaShi*) and 13% (*EW RaShi*). The *EW He* has the same mean dose to the right lung and target coverage as the reference plan. The integral delivered dose reduces for the *EW He* compared to the reference plan by 0.2%. For all plans, the contribution of helium to the total RBE‐weighted dose is less than 1%, being 0.57% (reference), 0.26%(*EW He*), 0.50% (*const RaShi*), and 0.48% (*EW RaShi*).

#### 3.3.3 Prostate case

The prostate case exhibits large residual helium ranges for some energy layers and gantry angles, with the residual range reaching up to 45cm at a gantry angle of 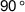. These large residual helium ranges complicate the detection. The *EW RaShi* method has the highest percentage of detectable spots. Still, for opposing beams (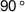, 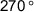) it is only 56% for the detector with the smallest sensitive range. For the detector with the biggest sensitive range, it increases to 88%. The other two investigated angles (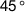, 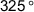) have a higher percentage of detectable spots. For the smallest detection system 72% can be detected, for the largest system this increases to 96%. In the prostate case, the residual helium range strategies selected distal range shifters.

#### 3.3.4 Liver case

For the liver case, the *EW RaShi* continued to show the highest percentage of detectable spots; up to 89% were detectable for a gantry angle of 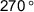 using the detector with the smallest sensitive range. Using the detector with the largest sensitive range enabled detection of 97% of the spots. Only for one gantry angle, 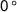, a distal range shifter was used.

### 3.4 Helium radiographs with range shifter

**FIGURE 11:**
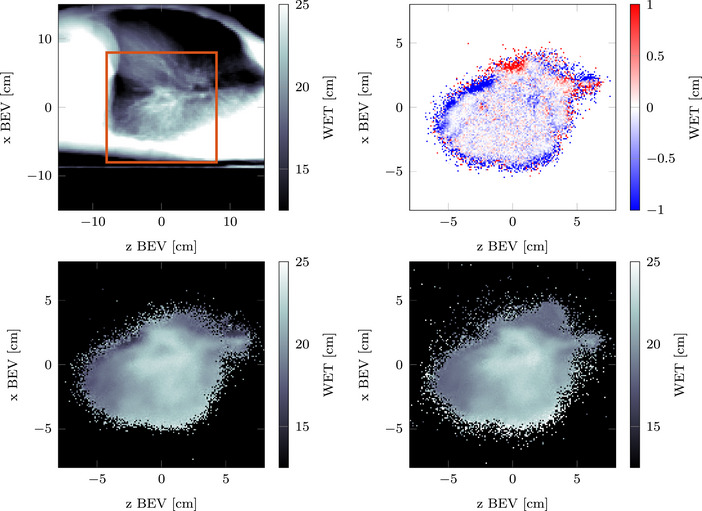
The top left shows the projected CT (for the 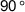 gantry angle), with the area highlighted (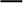) chosen for the mixed‐beam radiographs in the other subfigures. Bottom left: Simulated helium radiograph (197.58 ${\mathrm{MeVu}}^{-1}$, 0cm). Bottom right: Simulated helium radiograph (217.25 ${\mathrm{MeVu}}^{-1}$, 1.5cm). Top right: Difference image of both radiographs

In Figure 11, two helium radiographs are compared, one acquired from the reference plan and the other acquired from a *const RaShi* plan. The energies of the helium radiographs were chosen so that both plans, with and without the range shifter, irradiate approximately the same cross section of the tumor. The difference between both radiographs also indicates an increase in image noise when adding range shifters. When reconstructing the radiographs using ProtonVDA's reconstruction code, we use only primary helium ions and thus assume perfect particle identification. Therefore, the calculated noise increase does not account for the additional carbon or helium fragments detected. However, it does include the effects of increased scattering and range straggling.

## 4 DISCUSSION

This study used a mixed carbon–helium beam treatment planning framework to investigate the applicability and limits of the mixed beam method imposed by residual helium ranges at different patient sites, with a focus on lung patients. Residual helium range is a limiting factor in the selection of patients and treatment angles, as not all combinations are sensible without mitigating strategies. Two key considerations were identified: first, the helium ions must have sufficient range to exit the patient distally; second, the residual range should be in the sensitive range of the detector, as if the range is too high, the helium ions cannot be detected.

The analysis of the helium range, without any applied helium range strategy, showed insufficient helium range for at least one energy layer in almost all cases, independent of the gantry angle. The sensitive range of the employed detector was found to be a critical factor in ensuring good detection properties. This is particularly relevant for the prostate case, where the helium ions had a greater residual helium range, requiring a larger sensitive range.

For the lung cases, gantry angles of 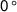 and 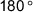 appear most suited for radiation therapy, as only a small portion of the spots exhibit insufficient residual helium range. These angles could also be combined in a two‐field treatment plan, where the upper region of the tumor is treated with a 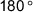 gantry angle, while the lower region is irradiated with a 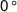 gantry angle, treating only spots with sufficient helium range from each angle. In the prostate case, a similar strategy could be used with opposing gantry angles of 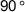 and 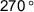. However, the limitation of this strategy is that only very few centers use carbon gantries like, for example, the HIT (Heidelberg Ion Therapy Center). Most facilities use fixed beam lines instead and are limited to horizontal and vertical orientations, with a few offering an oblique beam line^1^. A fixed beamline may offer advantages over a gantry for mixed‐beam irradiation, as it simplifies the setup of a distal range shifter. For greater flexibility in treatment angles, upright particle therapy^26^ would be beneficial. Upright therapy could also facilitate the use of a large detection system, as space in a gantry room is often limited, which would become particularly evident when a detector is combined with a distal range shifter.

For the lung sites with, in general, a smaller residual helium range, it seems that the smallest detector ($R^{maxD}$ = 11cm) is sufficient to enable good detection properties when a residual helium range strategy such as the *EW RaShi* is used. Under this approach, the mean increase of detectable spots between the detectors with 11cm and 16cm maximum detectable range was only 5 pp. However, the maximum increase of detectable spots between these detectors was 26 pp. Therefore, depending on the patient site and gantry angle, a detector with a larger sensitive range might still be sensible. For the detector with the largest investigated sensitive range ($R^{maxD}$ = 26cm), the median percentage of detectable spots was 78% (*EW He*), 100% (*const RaShi*), and 100% (*EW RaShi*). Another argument for using a detector with a larger sensitive range is that it allows for thinner distal range shifters, which in turn improves image quality. If a more complicated set‐up with a variable proximal and distal range shifter thickness were used, the additional degree of freedom should increase the percentage of detectable helium spots even more. However, the presented strategies using a simpler setup already allow for an increase of detectable spots.

As shown in Figure 8, when using the *const RaShi* strategy, the lowest energies may not be irradiated. Even with the selected proximal range shifter, the helium range may still be insufficient, and increasing the range shifter thickness would reduce the number of detectable spots. In most cases, the majority of spots lie within the central energy slices, with fewer spots in the high and low energy slices, so only a small number of spots are omitted. However, this omission can artificially favor the *const RaShi* strategy when evaluating the percentage of detectable spots, as some of the least favorable spots are not irradiated. Overall, each strategy results in a slightly different total number of spots in the treatment plan, as different energies are used.

The strategies, including range shifters, show a higher percentage of detectable spots, at the disadvantage of introducing additional scattering, leading to beam broadening and an increased total dose delivered to the patient. In our investigated patient plan, the use of range shifters led to an overall combined carbon–helium dose increase to the patient of 10% (Const RaShi) and 13% (EW RaShi) relative to the reference plan. The helium dose decreased, due to the now sufficient helium range, by 54% (EW He), 4% (Const RaShi), and 5% (EW RaShi) compared to the reference plan. Although the reference plan contained spots with insufficient helium range—resulting in dose deposition by helium ions with high LET at the end of their range—the total delivered dose remains lower. This trade‐off between the number of detectable spots and the total delivered dose may be of clinical relevance and should be evaluated more carefully in future work. In addition to increased scattering, the range shifters also lead to the production of more fragments. Furthermore, as illustrated in Figure 11, the use of range shifters increases image noise in the simulated radiographs. Experimental investigation of the detection properties, especially when thick distal range shifters are used is necessary.

The detectors used by Mazzucconi et al.,^1^ and Volz et al.^7^ for the experimental exploration of the mixed beam method covered a total WET of ∼127mm and ∼180mm, respectively. Our parameter selection was tailored to the ProtonVDA detector but also includes detectors with larger sensitive ranges up to 260mm. The sensitive WET ranges of other detection systems can be easily incorporated into the analysis, which can then be adapted to the specific system in use. As the analysis of the detection properties focuses on the residual helium range at the detector, it should be independent of the choice of ion‐counting versus integrating detector as used by Mazzucconi et al.,^1^ and Volz et al.^7^

While most detectors currently used in ion imaging are calorimeters or range telescopes, measuring residual energy or range, other detection systems are also used. Gehrke et al.^27^ used a thin silicon pixel detector for helium radiography, measuring deposited energy. By analyzing the cluster size and cluster volume, signals originating from primary helium ions can be identified. However, this system has a very sensitive WET range, making its application in mixed beam irradiation challenging The most accurate WET measurements are achieved in the steep gradient of the Bragg peak. Therefore, to accurately image complex objects, multiple energies are used.^28^

Another approach measures the time‐of‐flight (TOF) of an ion between two or more tracking units. From this measurement, the velocity and kinetic energy can be calculated. The sensitive WET range of such a detector depends on the distance between the tracking units and their time resolution.^29^, ^30^ For a TOF detector, the residual energy can be determined by measuring the TOF in air inside a TOF calorimeter distal of the patient. Additionally, the increase of an ion's TOF through the patient could be used as an indirect measure of energy loss, which could allow for particle identification.^30^ However, in a TOF system, the front tracer is crucial for accurate TOF measurements. Consequently, the high fluence in mixed‐beam irradiation poses significant challenges.

A detector designed for mixed beam radiotherapy should be capable of handling high particle fluxes and offer a large sensitive range. It should also provide particle identification to distinguish helium signals from those of fragments or the primary particles.

Simpler strategies for selecting the range shifter thickness were initially considered, such as basing it solely on the maximum residual helium range or on the patient's maximum WET. However, our proposed approach is designed to ensure adequate helium range while simultaneously maximizing the number of detectable spots. In the simplest configuration, we use a constant thickness throughout delivery (Const RaShi). A slightly more advanced approach allows the proximal thickness to vary (EW RaShi). To achieve this, we found that joint optimization of the proximal and distal shifter thicknesses is necessary. Selecting the proximal thickness based only on patient WET or the distal thickness based only on the maximum residual helium range is insufficient, because variations in the proximal thickness directly influence the residual helium range spectrum and, in turn, the optimal distal configuration.

In our investigation, we did not closely investigate the effect of the range shifter placement on image quality. The primary goal of this study was to identify generally valid combinations leading to detectable helium range, and as such the range shifters' thicknesses were the sensible free variables. Future work could more directly incorporate image quality metrics by including expected scattering and range straggling noise based on the transverse thickness in the selection algorithm Krah et al.^29^ and Collins‐Fekete et al.^31^ Our current investigation does not consider the dosimetric impact of each spot or the fluence of each spot and the corresponding signal‐to‐noise ratio. While these would be interesting metrics to incorporate into the selection process, we would expect the most distal regions of the tumor to exhibit the highest fluence,^32^ where at present, no range shifter or only a very thin one is used. The expected WEPL resolution could also be included, which would depend on the detection system—for example, whether a calorimeter or a multi‐stage range telescope is used, and if so, how many stages.^33^ Such a future analysis of the image quality could also optimize the weighting factor w in equation 4, which weights the number of detectable spots against the thickness of the range shifters.

## 5 CONCLUSION

The residual helium range in mixed beam treatment plans was evaluated across several beam angles in lung cases, as well as in a prostate and a liver case, revealing that the helium range was too low in most scenarios. Three different strategies were applied to ensure sufficient helium range. The *EW He* strategy uses a mixed carbon–helium beam only for energies with sufficient helium range, otherwise a pure carbon beam is used. The *const RaShi* and *EW RaShi* incorporate proximal and distal range shifters to ensure sufficient helium range while also optimizing detection capabilities by incorporating the sensitive range of the detector in the selection of the range shifters.

Overall, all evaluated residual helium range strategies successfully ensure sufficient helium range. Strategies involving range shifters further increase the number of detectable spots. These strategies allow more flexibility in the chosen treatment angle. All of this increases the clinical usability of the mixed carbon‐helium beam method, which shows promising potential for range verification, offering online beams‐eye‐view information, and allowing for the visualization of the treated patient's anatomy.

## CONFLICT OF INTEREST STATEMENT

The authors declare that they have no known competing financial interests or personal relationships that could have appeared to influence the work reported in this paper.

## Footnotes

1
https://www.ptcog.site/index.php/facilities‐in‐operation‐public
